# Biomaterial Constructs for Delivery of Multiple Therapeutic Genes: A Spatiotemporal Evaluation of Efficacy Using Molecular Beacons

**DOI:** 10.1371/journal.pone.0065749

**Published:** 2013-06-03

**Authors:** Jennifer C. Alexander, Shane Browne, Abhay Pandit, Yury Rochev

**Affiliations:** 1 Network of Excellence for Functional Biomaterials (NFB), National University of Ireland, Galway, Ireland; 2 National Centre for Biomedical Engineering Science, National University of Ireland, Galway, Ireland; INRS, Canada

## Abstract

Gene therapy is emerging as a potential therapeutic approach for cardiovascular pathogenesis. An appropriate therapy may require multiple genes to enhance therapeutic outcome by modulating inflammatory response and angiogenesis in a controlled and time-dependent manner. Thus, the aim of this research was to assess the spatiotemporal efficacy of a dual-gene therapy model based on 3D collagen scaffolds loaded with the therapeutic genes interleukin 10 (IL-10), a potent anti-inflammatory cytokine, and endothelial nitric oxide synthase (eNOS), a promoter of angiogenesis. A collagen-based scaffold loaded with plasmid IL-10 polyplexes and plasmid eNOS polyplexes encapsulated into microspheres was used to transfect HUVECs and HMSCs cells.The therapeutic efficacy of the system was monitored at 2, 7 and 14 days for eNOS and IL-10 mRNA expression using RT-PCR and live cell imaging molecular beacon technology. The dual gene releasing collagen-based scaffold provided both sustained and delayed release of functional polyplexes in vitro over a 14 day period which was corroborated with variation in expression levels seen using RT-PCR and MB imaging. Maximum fold increases in IL-10 mRNA and eNOS mRNA expression levels occurred at day 7 in HMSCs and HUVECs. However, IL-10 mRNA expression levels seemed dependent on frequency of media changes and/or ease of transfection of the cell type. It was demonstrated that molecular beacons are able to monitor changes in mRNA levels at various time points, in the presence of a 3D scaffolding gene carrier system and the results complemented those of RT-PCR.

## Introduction

Cardiovascular diseases are the leading cause of death in the Western world and account for more than 17 million deaths globally (WHO 2012) [1]. Cell-based therapies have been investigated to promote tissue regeneration, but have proven to be challenging due to cell death, low retention of cells at the site, and poor integration of cells with the native tissue [2]. Gene therapy is emerging as a potential therapeutic approach to address the challenges of cell-based strategies [3], with local gene transfer being a more effective therapy [4]. In addition, combination therapies are becoming increasingly important strategies to improve the efficacy of therapeutics [5], [6].

An appropriate gene therapy approach for cardiovascular pathogenesis may require multiple genes to enhance therapeutic outcome by modulating inflammatory response and angiogenesis in a controlled and time-dependent manner. Interleukin 10 (IL-10) is a potent multifunctional cytokine produced by a variety of cells [7]. It plays a crucial role *in vivo* in the attenuation of immune and inflammatory responses [8]. On the other hand, endothelial nitric oxide synthase is an inducible gene expressed in vascular endothelial cells (e.g. HUVECs) and few other cells [9], [10]. It is an enzyme that catalyses the conversion of the amino acid L-arginine to L-citrulline to produce nitric oxide (NO), a potent vasodilator and mediator of angiogenesis and arteriogenesis [11]. NO has multiple biological functions and plays an important role in cardiovascular homeostasis [12], [13].

Intracellular delivery of the genetic materials is the main challenge to specific and efficient gene therapy. There are two types of delivery systems available for gene transfer, viral and non-viral. Generally, non-viral vectors do not transfer gene material as efficiently as viral vectors [14]. However, non-viral vectors, typically plasmids, are considered safer as they generally exhibit lower toxicity, lower immune responses and do not integrate into the genome [15]. Among the agents used to form complexes with plasmid DNA and facilitate cellular uptake and transfection, SuperFect®, formulated from partially degraded (fractured) dendrimers, is one of the optimal [16]. Partially degraded dendrimers rupture endosomes to allow the escape of the plasmid DNA from degradation. Dendrimers are a class of polymers consisting of highly branched 3D macromolecules usually presenting well defined sizes and structures. The terminal groups exhibit high surface area presenting multiple sites for attachment of plasmids. These properties make dendrimers potential carrier candidates for gene delivery.

Plasmid DNA provides transient gene expression. However, sustained gene expression can be facilitated using biomaterial scaffolds which have the potential to maintain an effective level of the plasmid for the required time. Methods in which the plasmid DNA complexes are entrapped or encapsulated within the scaffold generally release complexes via the process of diffusion and scaffold degradation [17]. The loaded scaffold comes into close contact with target cells or tissues and enables localized delivery of the DNA that is released in a controlled and sustained manner.

Molecular beacons (MB) are short hairpin shaped oligonucleotide probes that are designed to hybridize their specific nucleic acid targets [18]. The complementary arms of the beacon forms a hybrid (the stem) which has a fluorophore dye and a quencher dye at either end in close proximity to keep the fluorescence of the beacon in the “off” state. Typically, the curved end (the loop) of the beacon hybridizes its target and the changes conformation separating the fluorophore from the quencher and fluorescence signal is emitted (the “on” state).

Molecular beacons (MB) can provide a spatiotemporal pattern of mRNA expression in living cells in real time within a short period (1–2 h) using delivery via reversible permeabilization with Streptolysin O [19], [20]. Thus, MB technology represents a quick and informative method to visualize the efficacy of gene-transfer from scaffolds. Also, MB can provide insight into localization pattern of mRNA expression levels in the individual cells transfected over a 14 day period.

The aim of this research was to assess the spatiotemporal efficacy of a dual-gene therapy model based on 3D collagen scaffolds using molecular beacons. Firstly, the scaffolding system was characterized to determine sustained and delayed release profiles over a 14 d period. Secondly, the spatiotemporal gene expression patterns for cells transfected with the scaffold were monitored at 2, 7, and 14 d using live cell imaging with molecular beacons. Thirdly, RT-PCR technique was used to quantify temporal gene expression and validate MB data.

In this study a gene therapy model for cardiovascular tissue engineering was evaluated. A dual gene releasing collagen-based scaffold loaded with pIL-10 polyplexes and peNOS polyplexes encapsulated into microspheres was used to transfect HUVECS and HMSCS. The therapeutic efficacy of the system over time was monitored for eNOS and IL-10 expression using RT-PCR and molecular beacon technology.

## Materials and Methods

### Ethics statement

HMSCs were isolated from the iliac crest of healthy donors after informed consent and approval by the Clinical Research Ethical Committee at University College Hospital, Galway.

### Cell culture

Human umbilical vein endothelial cells (HUVECs) were purchased from Lonza and cultured in endothelial basal medium (EBM-2) supplemented with the EGM-2 bullet kit (Lonza). Human mesenchymal stem cells (HMSCs) were isolated from the iliac crest of healthy donors under full ethical approval as previously described [21], [22] and were obtained from the Regenerative Medicine Institute, National University of Ireland-Galway. HMSCs were maintained in MEM α, medium (Gibco, Life Technologies, UK) supplemented with 10% research grade FBS (HyClone®, Thermo Scientific, UK), 1% penicillin/streptomycin (Sigma, 10,000 units ml^−1^) and 1 ng/ml fibroblast growth factor-2 (BD Biosciences, MA, USA).

### Molecular beacon design and synthesis

Molecular beacons labelled at the 5′-end with FAM fluorophore and the 3′end with BHQ 1 were designed to target a human eNOS or IL-10 mRNA following the protocol outlined in [23]. Briefly, the secondary (folding) structure of the target mRNAs were predicted using the Mfold web server, a software for nucleic acids folding and hybridization predictions [24], and single stranded regions were selected based on both ss-counts and p-num values. Then, OligoWalk software (Mathews Lab, University of Rochester Medical Center) [25] was used to select the optimal sequence that binds strongly to target mRNAs, and the sequence was queried using the BLAST database (basic local alignment search tool, National Center for Biotechnology Information) to ensure specificity for the target. A random control beacon that does not have any target in mammalian cells was included in the study. The sequences are shown in **Table 1**.

**Table 1:** Probes and primers design.

Molecular beacons	5′-3′ sequence
eNOS	FAM-**CACCGT**GTAGTACTGGTTGATGA**ACGGTG**-BHQ1
IL-10	FAM-**CGCAG**GGGAAGAAATCGATG**CTGCG**-BHQ1
Random	FAM-**CGACG**CGACAAGCGCACCGATA**CGTCG-**BHQ1

Primers	5′-3′ sequence
eNOS_forward	CTGAGAGACCAGCAGAGATACCAC
eNOS- reverse	CTGAAGCTCTGGGTCCTGAT
IL-10-forward	TGAGGCTACGGCGCTGTCAT
IL-10-reverse	TTCTTCACCTGCTCCACGGCCT
GAPDH-forward	GTCAGCCGCATCTTCTTTTGC
GAPDH-reverse	GCGCCCAATACGACCAAATC

Molecular beacon stem is indicated in **bold caps** and the **underlined** bases were shared with the probe on hybridization.

### Preparation of polyplexes

A human eNOS gene sequence encoded into a pcDNA3 vector containing the CMV promoter

was a kind donation from Dr. Karl McCullagh (Regenerative Medicine Institute, National University of Ireland-Galway). The plasmid encoding human IL-10, pORF-hIL10, was purchased from InvivoGEN (CA, USA). *Gaussia princeps luciferase* plasmids (GLuc; New England Biosciences, MA, USA) were labelled with Cy3 fluorophore using a Cy3 labelling kit (Mirus, WI, USA) as previously described [26]. Plasmids were combined with SuperFect® (3 mg/ml, Qiagen) to form polyplexes at ratio of SuperFect® and pDNA (µg∶µg) of 9∶1. The molar ratio of the nitrogen (N) of SuperFect® to the phosphate (P) of pDNA (N: P ratio) influences transfection efficiency and cytotoxicity and thus needs to be optimized for each cell type. A 6 µl volume of SuperFect® was mixed with 2 µg of plasmid DNA and allowed to form polyplexes for 5–10 min at room temperature.

### Fabrication of microspheres loaded with polyplexes

Hollow collagen microspheres were fabricated using a template method described elsewhere [26], [27]. Briefly, commercially available polystyrene beads (1 µm) (Gentaur, Chicago, Illinois) were sulfonated and coated with collagen at acidic pH for 4 h. The collagen coat was cross-linked using pentaerythritol poly(ethylene glycol) ether tetrasuccinimidyl glutarate (4S-PEG) for 2 h. The polystyrene core was dissolved to create hollow spheres by washing the coated beads with tetrahydrofuran (THF). Plasmid (2 µg) eNOS polyplexes were encapsulated into 1 µm hollow microspheres by agitation of the solution for 4 h, sterilized by the addition of 250 µl of absolute ethanol, and centrifuged at 13,000 rpm for 5 min. The supernatant was discarded.

### Fabrication of dual gene releasing collagen scaffolds

Collagen type I was extracted in the laboratory from bovine tendons using an acetic acid extraction method previously described elsewhere [28]. Briefly, a 5 mg ml^−1^ concentration was prepared using 0.5 M acetic acid. To prepare 4 mg ml^−1^ type I scaffolds, 5 mg ml^−1^ collagen was diluted on ice with 10X PBS, the pH adjusted to 7.0 using 2N NaOH. Solutions containing 2 µg plasmid IL-10 polyplexes were added to 2 µg plasmid eNOS-polyplexes encapsulated collagen microspheres before adding 100 µl of collagen solution and mixing gently by pipetting.

### Plasmid DNA release studies

Collagen scaffolds (see above) were prepared and loaded with Cy3 labelled GLuc-polyplexes or microspheres to compare their DNA release profile. Briefly, 100 µl of collagen solution (4 mg ml^−1^) was loaded with either Cy3 labelled GLuc-polyplexes or Cy3 labelled GLuc-polyplexes encapsulated into microspheres, and pipetted into 48-well plates (Nunc). After gelling for 30 min at 37°C/5% CO2, 300 µl of HBSS medium was added and plates were re-incubated and protected from light. Medium was replaced at time points from 1–14 d and collected medium was kept at −20°C until the final time point. A standard curve was prepared with quantification of plasmid DNA polyplexes released from the scaffold measured at excitation of 528 nm and emission ranging from 570–610 nm using the FLx800 Fluorescence Microplate Reader (BioTek,U.K).

### Gene delivery via collagen scaffolds

HUVECs and HMSCs were seeded into 8-well chambered cover glass (Nunc), 24-well glass bottom plates (MatTex Corporation, MA, USA) or 6-well plates the day before transfection experiment. The medium was removed and 25 µl (1 µg) or 100 µl (4 µg) of dual gene polyplexes loaded collagen mixture was added to each well and incubated for 30 min at 37°C/5% CO_2_ for gel formation. Untreated cells and cells treated with the “empty” collagen mixture without the polyplexes served as controls. After gelling, complete medium was added to each well and cells cultured for 2, 7, and 14 d before analysis for eNOS and IL-10 expression using RT-PCR and molecular beacon technology (live cell imaging). The media was changed every 2–3days for HUVECs and every 4 days for HMSCs.

### Cytotoxicity studies

HUVECs and HMSCs were seeded in 48-well plates (Nunc) at a density of 1×10^4^ cells per well the day before transfection experiments. Medium was removed and 25 µl of dual gene polyplexes (1 µg total) loaded collagen mixture was added to each well and incubated for 30 min at 37°C/5% CO_2_ for gel formation. Untreated cells (No scaffold control) and cells treated with the collagen mixture without the polyplexes (Empty scaffold control) served as controls. After gelling, complete medium was added to each well and cells cultured for 2, 7, and 14 d before alamarBlue™ assay was performed to determine cell metabolic activity, a measurement of cellular health.

### Molecular beacon detection of eNOS and IL-10 mRNA

HUVECs and HMSCs were seeded into 8-well chambered cover glass or 24-well glass bottom plates, respectively, at a density of 1×10^4^ cells per well. At 2, 7 and 14 d post gene transfection via 1 µg dual gene loaded collagen scaffolds (see above), cells were analysed for transcription of eNOS and IL-10. Molecular beacons targeting eNOS or IL-10 mRNA were delivered to cells via reversible permeabilization using Streptolysin O (SLO) as previously described [19]. Low concentrations of activated SLO can form pores in cell plasma membrane that can be repaired (resealed) by the addition of serum containing medium. Briefly, 2 U/ml SLO (Sigma) was activated with 5 mM TCEP (Sigma) in HBSS (without calcium or magnesium, Sigma) for 30 min at 37°C. Cells were incubated for 10 min with 100 µl of serum-free medium containing 0.2 U/ml SLO and 300 nM molecular beacons then rinsed three times with complete medium. Cells were incubated at 37°C for 1 h with 250 µl volume of complete medium to allow resealing of the plasma membranes before confocal imaging.

### RNA extraction and RT-PCR

HMSCs and HUVECs were seeded into 6-well plates at a density of 1×10^5^ cells/well before gene delivery via 4 µg-dual gene loaded collagen scaffolds (see above). Total RNA was isolated using 1 ml of Tri reagent® (Ambion) and purified using the RNeasy® Mini Kit (Qiagen) and on-column treated using DNase I (Qiagen). The purity and quantity of the RNA was determined using NanoDrop™ 1000 spectrophotometer (NanoDrop Technologies, DE, US). cDNA was obtained using 500 ng of total RNA and the Improm-II™ Reverse Transcription system with random primers (Promega, UK). Real-time PCR was performed using StepOnePlus™ Real Time PCR system (Applied Biosystems) and Fast SYBR Green PCR kit (Applied Biosystems). Relative quantification of eNOS and IL-10 was performed using the comparative C_T_ (crossing of threshold) method (ΔΔ C_T_ method) [29], [30] with human GAPDH as the internal control. IL-10 primers were designed using Primer-BLAST (National Center for Biotechnology Information) while previously published sequences for eNOS [31] and GAPDH [32] were used (**Table 1**). Each experiment was performed in triplicate. A 10 µl experiment contained 2 µl of diluted cDNA template, 1 µl of each 300 µM primer (the relevant reverse and forward primer), 1 µl of RNAse-free water, and 5 µl of Fast SYBR Green PCR mix.

## Results and Discussion

**Figure 1:**
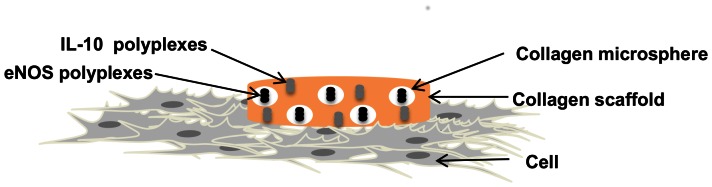
Illustration of the collagen-based system for dual gene delivery. Cells are seeded the day before the transfection experiment. A collagen scaffold loaded with IL-10 polyplexes and eNOS polyplexes encapsulated into collagen microspheres coats a portion of the cell monolayer. Polyplexes are released by degradation of the collagen (4 mg/ml) scaffold and collagen microspheres. Collagen microspheres were cross-linked to exhibit a more delayed degradation profile. IL-10 and eNOS mRNA expression levels were evaluated at 2, 7 and 14 d using RT-PCR and molecular beacons.

One of the aims of advanced biomaterials constructs is to deliver therapeutic molecules to cells in controlled and sustained manner. In particular cases where multiple genes are to be delivered, delayed release of one gene may be required. Our model sought to simultaneously deliver IL-10, (an anti-inflammatory cytokine) and eNOS (promotes angiogenesis) to cells. An illustration of the experimental system is shown in **Figure 1**.

### Characterization of the collagen scaffold delivery system

**Figure 2:**
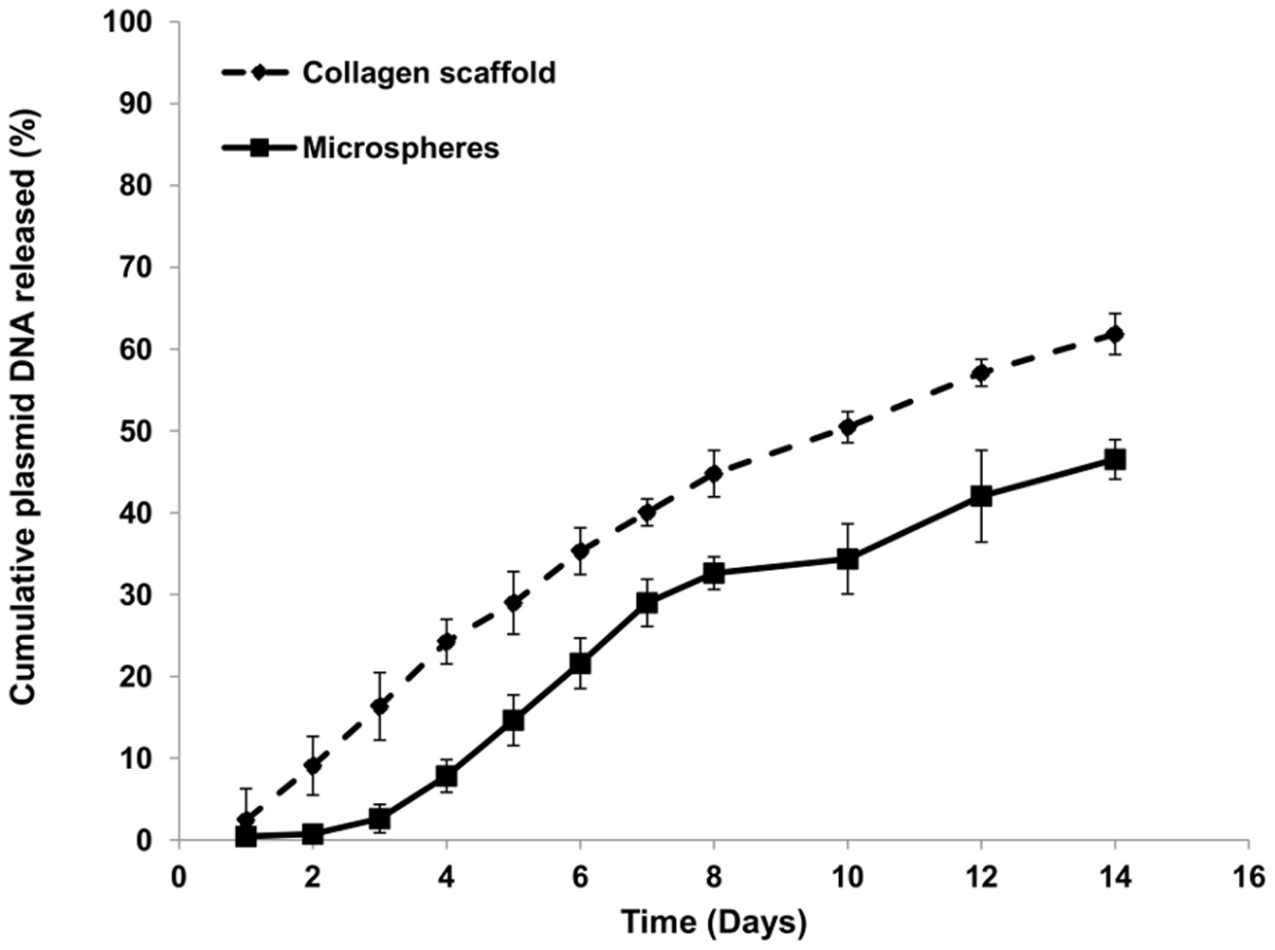
A representative plasmid DNA release over time in HBSS from polyplexes loaded collagen scaffolds. 2 µg of plasmid DNA polyplexes were loaded directly into the collagen scaffold or encapsulated in microspheres then loaded into the scaffold (microspheres). Media was changed at the time point indicated and the amount of Cy3 signal from the DNA quantified and calculated as a percent of the total DNA loaded. Data show mean ± standard deviation (n = 3).

In order for the therapeutic gene to be effective it has to be functional at the target site and remain there for the required period. Thus the release profile for polyplexes in the scaffold was monitored for 14 days. The ability of collagen scaffold system to provide both sustained and delayed delivery was assessed using Cy3-labelled pGLuc polyplexes. A sustained released was observed for polyplexes loaded directly into collagen scaffold which displayed an initial release of about 50 ng on day 1, followed by ∼125 ng/day from days 2–8 and 60 ng/day from days 9–14. Total plasmid DNA released from collagen scaffolds at day 14 ranged from 60–65%, as shown in **Figure 2**. Polyplexes encapsulated into collagen microspheres displayed a delayed release (<1 ng) over the first 2 days, followed by sustained released of ∼18 ng on day 3, ∼75 ng/day from days 4–8 and ∼30 ng/day from days 9–14. Total plasmid DNA released from collagen microspheres at day 14 ranged from 45–50%, as shown in **Figure 2**.

### Metabolic activity of transfected cells

**Figure 3:**
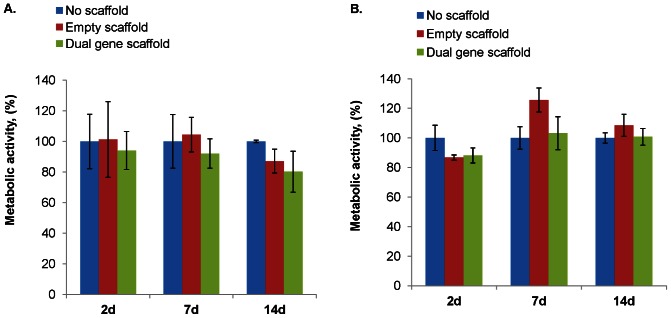
Metabolic activity in HUVECs (A) and HMSCs (B). The metabolic activity was measured for cells not treated with the scaffold or polyplexes (No scaffold), treated with the collagen scaffold not loaded with polyplexes (Empty scaffold) and treated with the collagen scaffold loaded with 0.5 µg each of plasmid IL-10 polyplexes and plasmid eNOS polyplexes encapsulated into microspheres (Dual gene scaffold) at 2, 7 and 14 d. The mean ± standard deviation are shown (n = 3 for each, p>0.05).

The cytotoxic effect of the dual gene system was evaluated in HUVECs and HMSCs using alamarBlue™ assay. “No scaffold” controls (cells only) and “Empty scaffold” controls (cells treated with the collagen scaffold not loaded with polyplexes) were prepared with each experiment. The dual gene scaffold did not have a significant effect on cell numbers since the metabolic activity in dual gene treated cells remains above 80% of the controls in both cell lines (**Figure 3**).

### Temporal expression levels of IL-10 and eNOS using RT-PCR

**Figure 4:**
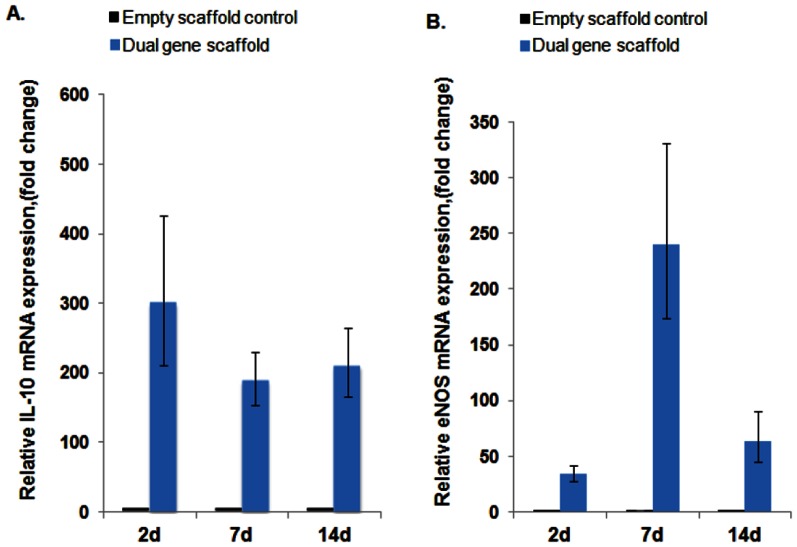
Fold changes in IL-10 (A) and exogenous eNOS (B) mRNA levels in transfected HUVECs. Cells were treated with collagen scaffold loaded with 2 µg each of plasmid IL-10 polyplexes and plasmid eNOS polyplexes encapsulated into microspheres (Dual gene scaffold) for 2,7, and 14 d. Control cells were treated with collagen scaffold containing no polyplexes or microspheres (empty scaffold control). Media was changed every 2-3 days. RT-PCR data showing the fold change in eNOS and IL-10 mRNA expression relative to 2 d control ± standard error of the mean are shown (n = 3 for each).

The efficiency of gene transfer from the dual-gene scaffolds was examined in primary HUVECs and HMSCs. Two controls were prepared with each experiment. The “No scaffold” control (cells not treated with the scaffold or polyplexes) was performed in order to assess scaffold related phenomena, while the “Empty scaffold” control (treated with the collagen scaffold not loaded with polyplexes) was performed to evaluate baseline mRNA levels. The IL-10 and eNOS mRNA expression in“No scaffold” controls were similar to “Empty scaffold” controls. To observe changes due to dual gene-transfer from the scaffolds loaded with both the 2 µg polyplexes, IL-10 and eNOS mRNA expression levels were calculated relative to the day 2 “Empty scaffold” control. Differences in IL-10 and eNOS mRNA expression levels were observed between the two cell types. IL-10 mRNA levels in transfected HUVECs were slightly higher at day two compared to day seven and 14 (**Figure 4A**). This is probably due to the lower concentrations of IL-10 polyplexes available to cells due to twice as much media changes compared to HMSCs. It appears that diffusion is the prominent process of release of the IL-10 polyplexes from the non-cross-linked collagen scaffold. The expression levels of eNOS mRNA in transfected HUVECs were highest at day seven and lowest at day two (**Figure 4B**). The slower release of polyplexes from cross-linked collagen microspheres may have contributed to the increase in eNOS mRNA expression levels seen at day seven and 14.

**Figure 5:**
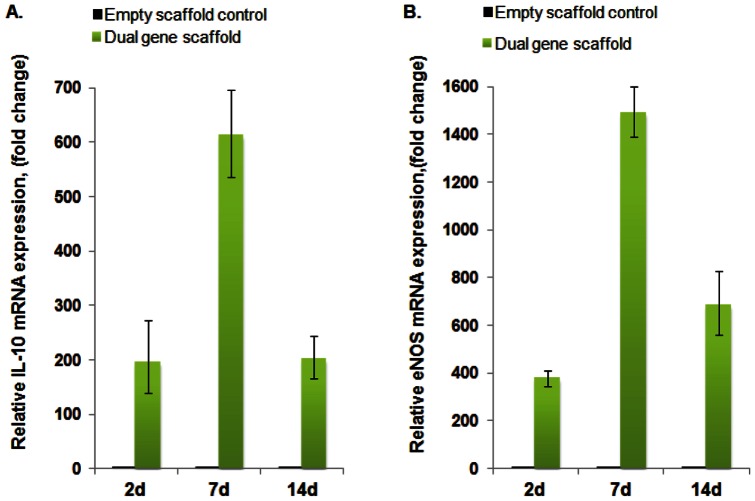
Fold changes in IL-10 (A) and exogenous eNOS (B) mRNA levels in transfected HMSCs. Cells were treated with collagen scaffold loaded with 2 µg each of plasmid IL-10 polyplexes and plasmid eNOS polyplexes encapsulated into microspheres (Dual gene scaffold) for 2,7, and 14 d. Control cells were treated with collagen scaffold containing no polyplexes or microspheres (empty scaffold control). Media was changed every 4 days. RT-PCR data showing the fold change in eNOS and IL-10 mRNA expression relative to 2 d control ±standard error of the mean are shown (n = 3 for each).

On the other hand, IL-10 mRNA levels in HMSCs transfected with dual-gene scaffolds were highest at day seven while day two and 14 levels were similar (**Figure 5A**). Also, the expression levels of eNOS mRNA after gene scaffold transfection were highest at day seven -and lowest at day two (**Figure 5B**). The levels of eNOS and IL-10 mRNA from transfected HMSCs and HUVECs were significantly different to empty scaffold controls which contained no polyplexes, and may indicate quiescent levels of these mRNAs in the control cells [33], [34]. While in HUVECs it appears that eNOS plasmid release from dual gene scaffolds is delayed compared to IL-10, this did not seem to be the case in HMSCs as the expression of eNOS has an effect on endothelial cell function more so than that seen in HMSCs [35], [36].

### Spatiotemporal expression patterns of IL-10 and eNOS mRNA using MB

**Figure 6:**
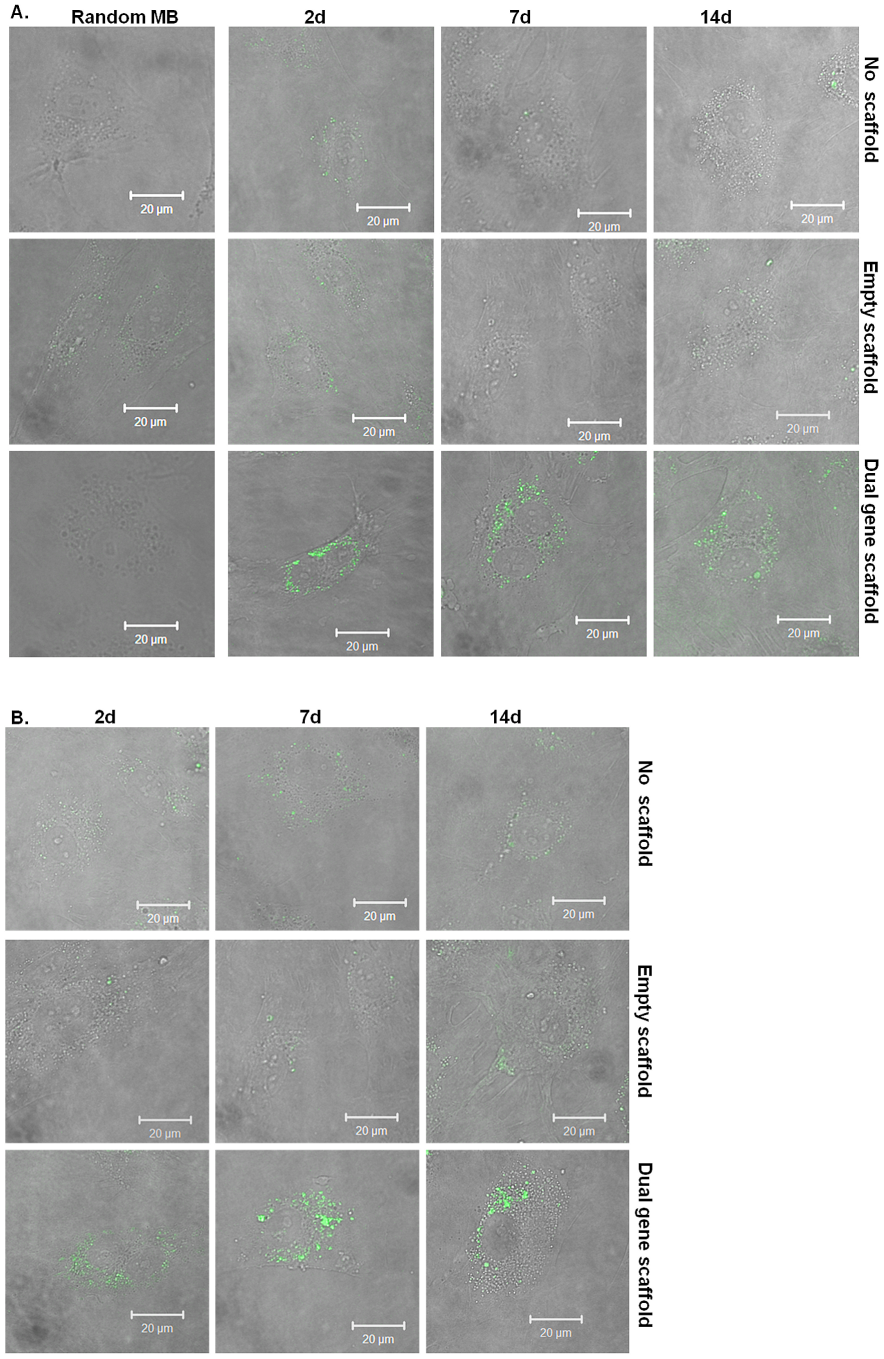
Detection of IL-10 (A) and eNOS (B) mRNAlevels in transfected HUVECs using 300 nM MBs. FAM fluorescence signal from IL-10 (A) and eNOS (B) MBs in cells not treated with the scaffold or polyplexes (No scaffold), treated with the collagen scaffold not loaded with polyplexes (Empty scaffold) and treated with the collagen scaffold loaded with -0.5 µg each of plasmid IL-10 polyplexes and plasmid eNOS polyplexes encapsulated into microspheres (Dual gene scaffold) at 2, 7 and 14 d.

To evaluate the spatiotemporal efficacy of IL-10 and eNOS gene-transfer to HUVECs and HMSCs molecular beacons were delivered to the cells using SLO mediated delivery. In this experiment 0.5 µg each of IL-10 polyplexes and encapsulated eNOS polyplexes were loaded into the scaffold and delivered to cells (∼10,000) in monolayer. “No scaffold” controls and “Empty scaffold” controls were prepared to evaluate collagen scaffold related phenomena. The presence of the collagen scaffold seemed to slightly increase baseline levels of IL-10 and eNOS mRNA expression in HUVECs more so than in HMSCs. IL-10 mRNA signals were observed in HUVECs cells in all treatment groups, and transfected cells seemed to exhibit increased signal intensities compared to control cells (**Figure 6A**). Changes in signal intensity between days 2 to 14 were not evident with localization pattern that seems to be concentrated in perinuclear and ribosomal regions of the cell (**Figure 6A**). No signal was observed with control (random) beacon which has no targets in the cells, suggesting the specificity of the IL-10 MB. The signal intensity from eNOS MB in transfected HUVECs showed low signal at day two which increased by day seven and 14 (**Figure 6B**). Signal localization for eNOS mRNA appears to be perinuclear and at times concentrated to one region of the cell (probably associated with the Golgi) which is more evident at day 14.

**Figure 7:**
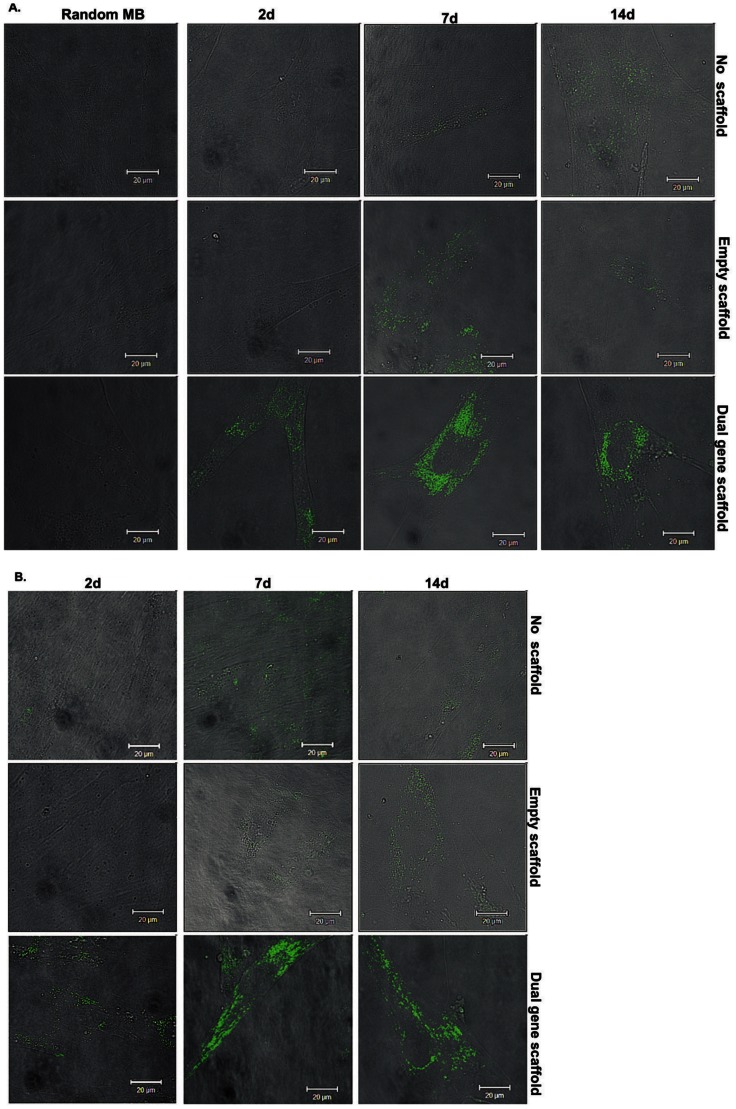
Detection of IL-10 (A) and eNOS (B) mRNA levels in transfected HMSCs using 300 nM MBs. FAM fluorescence signal from IL-10 (A) and eNOS (B) MBs in cells not treated with the scaffold or genes (No scaffold), treated with the collagen scaffold not loaded with genes (Empty scaffold) and treated with the collagen scaffold loaded with 0.5 µg each of plasmid IL-10 polyplexes and eNOS polyplexes encapsulated into microspheres (Dual gene scaffold) at 2, 7 and 14 d.

IL-10 and eNOS MB signals from transfected HMSCs appeared more intense than controls (**Figure 7A** & **Figure** **7B**). Signal intensities in HMSCs varied at the different time points with low signal at day two and higher signal intensities at day seven and 14. In HMSCs, the localization pattern for eNOS mRNA appeared distributed throughout the cytoplasm, while IL-10 mRNA showed localization in the perinuclear and ribosomal regions.

### Comparison of RT-PCR and molecular beacons technology

Both RT-PCR and MB data for IL-10 mRNA expression corresponded well with each other. The sequence of the eNOS plasmid used for gene-transfer has 85% homology with a human eNOS mRNA which made the selection of primers that detected total eNOS mRNA impossible. MB was used to detect both total (endogenous and plasmid) eNOS, while the RT-PCR primers were specific for the plasmid eNOS. Both RT-PCR and MB data demonstrated low levels of eNOS mRNA in transfected cells at day two which was increased at day 7.

## Conclusions

DNA-polyplexes encapsulated into collagen microspheres or directly loaded into collagen scaffolds remained functional and were able to transfect cells. Further, encapsulation of eNOS DNA polyplexes into cross-linked collagen microspheres resulted in delayed release of this gene to the cells. This was evident from eNOS MB images which showed low FAM fluorescence signal at day two in both transfected HUVECs and HMSCs, confirmed by RT-PCR data which showed the lowest eNOS mRNA expression at day two. IL-10 mRNA expression for HUVECs remained at relatively constant level, while that of HMSCs was increased to a maximum at day seven and decreased to day two levels by day 14. This may be due to less IL-10 polyplexes being available to HUVECs as a result of twice as much media changes compared to HMSCs along with the faster IL-10 release profile. This decrease may also be due to lower transfection efficiency in HUVECs. The decrease in mRNA expression at day 14 could be explained by decreased concentration of polyplexes available to transfect an increased cell population (doubling time about 4 d) and the transient expression of plasmid transfection (transfected cells undergo mitosis and dilution occurs as plasmid DNA is not passed on to daughter cell, or is degraded). However, the collagen scaffolds (4 mg/ml) loaded with dual genes can provide sustained delivery of these genes for up to 14 d as noted by the 50–1400 fold increased in mRNA from day 2–14 when compared to controls. It was demonstrated that molecular beacons are able to monitor changes in mRNA levels at various time points, in the presence of 3D a scaffolding gene carrier system and the results confirmed by RT-PCR.
